# Rapid Prediction of Fig Phenolic Acids and Flavonoids Using Mid-Infrared Spectroscopy Combined With Partial Least Square Regression

**DOI:** 10.3389/fpls.2022.782159

**Published:** 2022-03-10

**Authors:** Lahcen Hssaini, Rachid Razouk, Yassine Bouslihim

**Affiliations:** National Institute of Agricultural Research (INRA), Rabat, Morocco

**Keywords:** FTIR-ATR, partial least square regression (PLSR), *Ficus carica* L., phenols, HPLC-DAD

## Abstract

Mid-infrared spectroscopy using Fourier transform infrared (FTIR) with attenuated total reflectance (ATR) correction was coupled with partial least square regression (PLSR) for the prediction of phenolic acids and flavonoids in fig (peel and pulp) identified with high-performance liquid chromatography-diode array detector (HPLC-DAD), with regards to their partitioning between peel and pulp. HPLC-DAD was used to quantify the phenolic compounds (PCs). The FTIR spectra were collected between 4,000 and 450 cm^–1^ and the data in the wavenumber range of 1.175–940 cm^–1^, where the deformations of O-H, C-O, C-H, and C=C corresponded to flavanol and phenols, were used for the establishment of PLSR models. Nine PLSR models were constructed for peel samples, while six were built for pulp extracts. The results showed a high-throughput accuracy of such an approach to predict the PCs in the powder samples. Significant differences were detected between the models built for the two fruit parts. Thus, for both peel and pulp extracts, the coefficient of determination (R^2^) ranged from 0.92 to 0.99 and between 0.85 and 0.95 for calibration and cross-validation, respectively, along with a root mean square error (RMSE) values in the range of 0.46–0.9 and 0.23–2.05, respectively. Residual predictive deviation (RPD) values were generally satisfactory, where cyanidin-3,5-diglucoside and cyanidin-3-O-rutinoside had the higher level (RPD > 2.5). Similar differences were observed based on the distribution revealed by partial least squares discriminant analysis (PLS-DA), which showed a remarkable overlapping in the distribution of the samples, which was intense in the pulp extracts. This study suggests the use of FTIR-ATR as a rapid and accurate method for PCs assessment in fresh fig.

## Graphical Abstract

Scheme diagram showing the research methodology and analytical approaches.

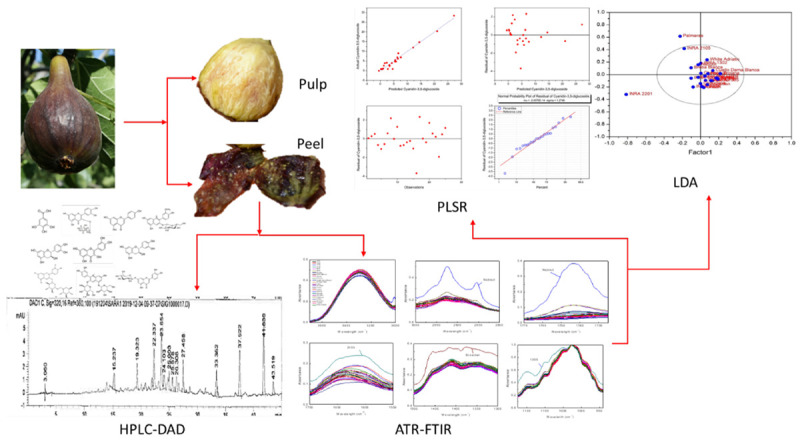

## Introduction

The rapidly growing interest in functional foods, particularly underutilized fruits and beverages is based on their uniqueness as natural bioactive resources, necessary to enhance the human health and well-being. Worldwide, large species are not fully assessed for their nutritional components and biologically active compounds involved in consumer health promotion so far. Although the naturally occurring phenotypic, chemotypic, and ecotypic diversity of most of these species is still scarcely screened, it is evident that they present an invaluable potential source of bioactive compounds directly associated with the prevention of coronary diseases. Particular attention should be devoted to the investigation of these species’ bioactive compounds, mainly their secondary metabolites, since they not only present the main quality indicators of new cultivars but are also important in chemotaxonomy (Česoniene et al., 2009). Phenolic acids and flavonoids are among the most chemically heterogeneous groups of secondary metabolites synthetized by plants. Screening of these bioactive molecules has gained the most interest during these decades, since it helps in recognizing new raw materials for food, nutraceutical, and cosmetic industries. Figs are an emblematic food of the Middle Eastern and North African diets and constitute an important source of phenolic compounds (PCs) which contribute significantly to their taste, color, astringent flavors, and aroma (Vinson et al., 1998; Kamiloglu and Capanoglu, 2013; Haytowitz et al., 2018; Arvaniti et al., 2019). Anthocyanins, particularly cyanidin-3-rutinoside; flavanols, mainly quercetin-rutinoside; phenolic, such as chlorogenic acid; and flavones, such as luteolin and apigenin-rutinoside, were reported as the major PCs isolated from fresh figs (Vallejo et al., 2012; Viuda-martos et al., 2015; Harzallah et al., 2016).

For high quantification accuracy of these compounds, high performance liquid chromatography (HPLC), with diode array detector (DAD) or even coupled with mass spectroscopy (MS), are among the frequently used conventional techniques (Nadeem and Zeb, 2018). However, these methods are time-consuming, expensive, and require laborious work and a large number of solvents, some of which are hazardous (Koch et al., 2014). Therefore, accurate, rapid, with minimal sample preparation, and low-cost technologies are highly required. Fourier transform infrared (FTIR) spectroscopy presents all these features and therefore, has become among promising spectroscopic technologies, widespread in the analysis of main food components. This technology is highly sensitive, providing different levels of molecular information regarding primary and secondary metabolite structures (Bouyanfif et al., 2019). It measures predominantly molecular vibration and determines its intensity by the dipole moment change during the vibrational mode and provides a specific infrared spectrum, which is the biochemical fingerprint that provides information about molecular composition (Nadeem and Zeb, 2018). Earlier studies showed that the FTIR spectroscopy provided satisfactory results with high-throughput screening framework of numerous biomolecules in several food samples mainly fruits, vegetables, or beverages, e.g., biosynthesis of silver nanoparticles in figs (Jacob et al., 2017), Sudanese honey (Tahir et al., 2017), discrimination of bovine, porcine and fish gelatins (Cebi et al., 2016), fatty acid changes in *Caenorhabditis elegans* (Bouyanfif et al., 2019), mini kiwi (Baranowska-Wójcik and Szwajgier, 2019), red bell pepper (Prabakaran et al., 2017). Therefore, FTIR spectroscopy has emerged as a potential alternative for highly rapid metabolic fingerprinting technique, which can be applied in conjunction with an attenuated total reflection (ATR) correction procedure (Mamera et al., 2020). However, FTIR provides complex spectra, which consists of many related variables (wavenumber) per sample, making its visual analysis very difficult. Hence, the chemometric and data mining approaches are usually applied to simplify or dimensionally reduce the dataset to fewer independent parameters, with a minimal loss of total variance, thereby making human interpretation easier (Lu et al., 2011). Achieving reproducible results, spectra dimensionality reduction and high fingerprinting throughput acquisition requires a rigorous use of chemometric models capable of associating the numerous spectral intensities from multiple calibration samples to identify chemical fingerprints within samples by removing potential outliers and determining the principal components capturing the high amount of total variance within the dataset (Mamera et al., 2020). The most commonly used multivariate calibrations is partial least squares (PLS), which is an appropriate method for predistortion when highly collinearity is present within the dataset.

One generally agreed, the chemometrics of multivariate adjustments may be used efficiently to identify the relationships between the actual concentration of targeted compounds, as determined by conventional methods, such as HPLC-DAD and predicted amounts using the mid infrared spectroscopy (FTIR spectroscopy in this case) (Tejamukti et al., 2020). Partial least squares regression (PLSR) is a versatile discriminant method, that has been well documented (Brereton and Lloyd, 2014; Mehmood and Ahmed, 2016; Lee and Jemain, 2019). Mathematically, the PLS model has a similar approach to PCA to project high dimensional data into a series of linear subspaces of the explanatory variables (Lee and Jemain, 2019). However, the PLS model assumes a supervised learning process instead of an unsupervised learning approach in the PCA model (Yang and Yang, 2003). The application of FTIR spectroscopy and HPLC combined with multivariate calibration for analysis was reported over several biological raw materials, showing a very significant prediction accuracy, such as honey (Anjos et al., 2015; Tahir et al., 2017), virgin olive oil (Hirri et al., 2016), Mangosteen (Tejamukti et al., 2020), grape, carob and mulberry (Yaman and Velioglu, 2019), coffee beans (Liang et al., 2016), salvia seeds (Tulukcu et al., 2019), and kakadu plum powders (Cozzolino et al., 2021).

Being the third worldwide fig producer, Morocco recognizes this fruit as a principal component of the local people diet. Despite hosting a wide range diversity of this species, local figs remain poorly screened for their biochemical fingerprints linked to its nutritional quality, mainly because of the classic methods used so far are often costly. In this context, the application of rapid, efficient, and the sampling cost approach, with approved prediction accuracy, is of utmost required. In this study, we aimed at screening twenty-five fig cultivars using HPLC-DAD and attenuated total reflectance Fourier transform infrared (ATR–FTIR) coupled with PLS. This study was designed to assess the fig peels and pulps of sampled cultivars separately to evaluate the throughput resolution of PCs prediction through the abovementioned design over the two fruits parts. It also attempts to determine which part of the fruit provides the most relevant discrimination among cultivars. This is the first attempt to predict the amounts of phenolic acids and flavonoids through modeling a colossal FTIR-ATR spectral dataset of fig peels and pulps in Morocco. Building those models for eventual confirmation through time, will immensely contribute to manage the wide range of fig diversity hosted in Moroccan agroecosystems, as a first screening stage with time saving and satisfactory precision level. This study will serve as a useful reference for researchers, food and nutraceutical industries, helping to efficiently and rapidly assess these key fingerprints.

## Materials and Methods

### Plant Material and Experimental Design

Figs of an *ex situ* collection were randomly harvested at their complete ripening stage. The collection is composed of 16 local and 9 exotic varieties which have been planted in 2005 in the experimental station of the National Institute for Agricultural Research of Meknes (INRA) in the Northern Morocco. The collection is conducted in a complete randomized block on ferritic soil. These cultivars were chosen based on our previous studies on 135 cultivars under the same conditions and experimental design, which were screened for their chemotypic (Hssaini et al., 2019) and morphotypic (Hssaini et al., 2020a) diversity besides the combination of both (Hssaini et al., 2020b). The findings of the aforementioned studies revealed highly significant variability, where the cultivars herein investigated captured a high level of total variance and underwent further analysis. Table 1 shows the fig cultivar herein investigated, their geographical origin, ripening period, and growing conditions along with their respective codes. Figs were considered fully ripened when they were easily separated from the twig and when the receptacle turned to reddish-purple coloration.

**TABLE 1:** Cultivars geographical origins, harvest time, and monthly meteorological data from August to early September 2018 in Northern Morocco, Meknes (Ain-Taoujdate experimental station -INRA).

	Cultivars	Code	Geographical origin	August						September	
				[1–5]	[6–10]	[11–15]	[16–20]	[21–25]	[26–30]	[31–4]	[5–9]
Local	El Quoti Lbied	G13	Morocco								
	Nabout	G18									
	Fassi	G14									
	Noukali	G19									
	Ghoudan	G15									
	Chetoui	G11									
	Bioudie	G7									
	Chaari	G10									
	Ournaksi	G20									
	INRA 1305	G3									
	INRA 2105	G4									
	INRA 1302	G2									
	INRA 2201	G5									
	INRA 2304	G6									
	INRA 1301	G1									
Introduced	Snowden	G23	United States								
	White Adriatic	G25	Italy								
	Kadota	G17	Italy								
	Troiana	G24	Italy								
	Cuello Dama Blanca	G12	Spain								
	Breval Blanca	G9	Spain								
	Palmeras	G21	Spain								
	Herida	G16	Spain									
	Breba Blanca	G8	Spain								
Total rainfall (mm)				0	0	0	0	0	26.4	0	0
Average temperature (°c)				25.84	28.5	27.56	29.24	29.44	23.64	25.6	25.42
Average solar radiation (W/m^2^)				169.29	208.74	243.83	238.28	185.35	123.5	270.21	271.38
Soil type				Sandy clay loam with an average organic matter of 1% [0–30 cm soil layer]							
Soil pH				7.2							

*Climatic data collected from meteorological station installed next to the orchard.*

### Samples Preparation

Immediately after harvesting, the fruits were manually peeled using a sharp stainless knife and both peels and pulp inclosing seeds were sliced, frozen under −80°C for 48 h and then lyophilized at a pressure of 0.250 mbar and temperature for −55°C for 48 h (Alpha 1-2 LD_plus_ lyophilizer, Christ, Osterode, Germany). Hereto, triplicate lots of fig fruits from each genotype were grounded to a powder using an IKA A11 Basic Grinder (St. Louis, MO) at room temperature. The samples were then packaged in polyethylene terephthalate (PET) bags (size: 17 cm × 12 cm L/W; permeability: 50–100 and 245.83–408.64 cm^3^ μm/m^2^ h atm for O_2_ and CO_2_, respectively; permeability to water vapor: 16.25–21.25 g μm/m^2^ h) and vacuum sealed and then, kept refrigerated at 4°C until FTIR-ATR and HPLC-DAD fingerprinting.

### Fourier Transform Infrare Spectroscopy

Fourier transform infrared spectra of fig peels and pulps powders were collected between 4,000 and 450 cm^–1^ at a resolution of 4 cm^–1^ on Perkin–Elmer Fourier transform infrared spectrometer (Perkin Elmer, Waltham, MA, United States). At room temperature, each sample was scanned three times in distinct randomly mass (50 mg) of the sample. For each FTIR spectrum, three scans were averaged and IR spectrum corresponded to the accumulation of 128 scans. The germanium crystal was in contact with the sample after applying a pressure setting at a maximum of 1,700 kg/cm^2^ to ensure uniform distribution of the sample across the crystal and to achieve a high-resolution of the acquired IR spectra (Szakonyi and Zelkó, 2012). Prior to sample measurements, a background spectrum was collected from an empty germanium crystal surface and automatically subtracted from the spectra of the sample. The crystal cell was cleaned between spectral collections using ethyl alcohol and warm water and dried with absorbent paper. The standard normal variate (SNV) and multiplicative scattering correction (MSC) were first carried out to correct multiplicative interferences (Tahir et al., 2017). Then, the raw FTIR spectra were corrected by the extended ATR correction procedure using Essential FTIR software (version 3.50.183) (angle of incidence = 45 degrees; number of ATR reflection = 1; mean refractive index of sample = 1.5; maximum interaction = 50; 1.8 mm crystal surface). The most important feature of ATR is the evanescent field, which occurs during the reflection of IR light at the interface of a material with a high refractive index (ATR crystal) and a material with a low refractive index (sample) (de Nardo et al., 2009). The full spectra of fig samples indicate the presence of some regions corresponding to the sample fingerprints of which integrated areas were measured using Essential FTIR software.

### Determination of Phenolic Compounds

#### Extraction Method

For each sample, 1 g of peel and pulp powder were separately mixed with 10 ml of methanol:water (80:20, v/v). The mixture was sonicated using an ultra-sonicator UP 400St Hielscher’s (400 W, 24 kHz) and then macerated for 60 min at 4^°^C. Afterward, it was centrifuged for 10 min, 8,000 g at 4^°^C (Eppendorf Centrifuge 5804, Eppendorf, Hamburg, Germany) and the supernatant was collected and the sediment was mixed with 10 ml of acetone:water (70:30, v/v). The same steps (sonication, maceration, and centrifugation) were repeated three times, and the supernatants were mixed together and then evaporated using a rotary evaporator (Büchi R-205, Switzerland) under a speed of 1,500 rpm and reduced pressure, at 40^°^C. Then, 5 ml of methanol was added to the residue, and the mixture was well shaken in a Vortex for 2 min. The samples were filtered through a Sep-Pak (c-18) to remove the sugar content and then were stored at −20^°^C until further use.

#### Phenolic Compounds Assessment

Polyphenolic profiles of both peel and pulp fruits were determined by HPLC as described by Genskowsky et al. (2016). Briefly, a volume of 20 μl of each sample was injected into a Hewlett-Packard HPLC series 1200 instrument equipped with C18 column (Mediterranea sea 18, 25 cm × 0.4 cm, 5 cm particle size) from Teknokroma (Barcelona, Spain). Polyphenolic acids and flavonoids were assessed in standard and sample solutions, using a gradient elution at 1 ml/min. The mobile phases consisted of formic acid in water (1:99, v/v) as solvent A and acetonitrile as solvent B. The chromatograms were recorded at 280, 320, 360, and 520 nm. A quantitative analysis of PCs was carried out by reference to authentic standards: gallic acid, (+)-catechin, (−)-epicatechin, chlorogenic acid, quercetin-3-O-rutinoside, quercetin-3-O-glucoside, luteolin-7-O-glucoside, quercetin, apigenin, cyanidin-3,5-diglucoside, cyanidin-3-O-rutinoside, and pelargonidin-3-O-rutinoside (Extrasynthese, Genay, France). Their identification was carried out by comparing the UV absorption spectra and retention times of each of them with those of pure standards injected under the same conditions. Each sample was assessed in triplicate and the results were expressed as μg/g of the dry weight (dw).

The linearity of the method above described was evaluated by analyzing the herein used standard solutions at different concentrations. An average correlation coefficient of 0.987 was obtained through calibration curves for all the standards. Afterward, the recovery test was performed by spiking samples at different concentrations with known amounts of each standard. Spiked and unspiked extracts were then analyzed in triplicate. With regards to the complexity of the samples, satisfactory recovery levels were obtained (86–98%) alongside low standard error values within a narrow range of variation (0.07–1.23%).

### Data Processing

Prior to statistical analysis, data were tested for normality and homogeneity. Afterward, one-way ANOVA was performed using SPSS software V 22 to test significant differences among the samples (*p* < 0.05) in both peels and pulps. The principal component model was performed to detect any spectral outliers in the FTIR-ATR data prior to build a prediction model using PLSR. Interference and overlapping in the obtained spectra may be overcome by using a powerful multicomponent method, such as PLSR (Hirri et al., 2016). Therefore, PLSR was used for building predictive models between the FTIR-ATR spectra and HPLC-DAD reference data for both fig peel and the pulp using OriginPro software v 9 (OriginLab Corporation Inc.). The optimum numbers of factor to be extracted were decided based on the model with minimum root mean predicted residual sum of squares (PRESS) by jack-knifing within a cross-model validation framework to inspect the predictive capability of all calibration models. Jack-knifing is a cross validation procedure that relies on uncertainty tests of the regression coefficients to test the significance of the model parameters (Karaman et al., 2013). The samples were randomly divided into two subsets. One of them was used to develop a model (calibration set = 20 samples) and the second one was used to validate the robustness of the built model (prediction set = 5 samples). According to Goodhue et al. (2006) and Sarstedt et al. (2017), PLSR can be applied efficiently on a small sample size particularly when models are complex. Besides, many previous studies have performed a PLS regression model on smaller sample size with satisfactory results (Tenenhaus et al., 2005; Zheng et al., 2017). The accuracy of PLSR models was assessed in the terms of root mean square error (RMSE), the correlation coefficient (*R*^2^) between actual and predicted values along with the residual predictive deviation (RPD), which is calculated as the ratio of the SD of the dependent data to RMSE (Cozzolino et al., 2021). PLSR models were performed using the FTIR-ATR data within the vibration region of 1,175–940 cm^–1^.

## Results and Discussion

### Phenolic Profile

Table 2 shows the descriptive statistics (average, range, and SD) along with the ANOVA for the concentration of phenolic fractions in the fig peel and pulp of investigated cultivars. High performance chromatography analysis identified several PCs belonging to phenolic acids (hydroxycinnamic acid and hydroxybenzoic acid derivatives) and flavonoids (flavonols, flavones, and anthocyanidins). Eight PCs were identified over the pulp samples, mainly (+)-catechin, (−)-epicatechin, chlorogenic acid, quercetin-3-O-rutinoside, quercetin-3-O-glucoside, luteolin-7-O-glucoside, cyanidin-3,5-diglucoside, and cyanidin-3-O-rutinoside. On the other hand, twelve PCs were isolated: gallic acid, (+)-catechin, (−)-epicatechin, chlorogenic acid, quercetin-3-O-rutinoside, quercetin-3-O-glucoside, luteolin-7-O-glucoside, quercetin, apigenin, cyanidin-3,5-diglucoside, cyanidin-3-O-rutinoside, and pelargonidine-3-O-rutinoside. These compounds displayed highly significant differences across cultivars following both fruits parts (*p* < 0.001) (Table 2). Among all sampled fruits, the PCs concentrations were higher in peels compared with pulps extracts. Anthocyanins, particularly cyanidin-3,5-diglucoside and cyanidin-3-O-rutinoside, were predominant in peel extracts, of which the average concentrations were 75.902 ± 18.76 and 77.972 ± 18.95 μg/g dw, respectively. Regarding flavonols, only (−)-epicatechin, quercetin-3-O-rutinoside, and quercetin-3-O-glucoside were identified. Gallic acid and pelargonidin-3-O-rutinoside were only detected in two cultivars “Chetoui” and “Nabout,” with the respective levels of 8.363 ± 1.88 and 6.731 ± 2.019 μg/g dw. These results agree with those reported in previous research (Vallejo et al., 2012; Hirri et al., 2016; Cozzolino et al., 2021). Only one sample (‘1301’) has displayed the highest levels in almost all identified compounds, especially (−)-epicatechin, quercetin-3-O-rutinoside, quercetin-3-O-glucoside, cyanidine-3,5-diglucoside, and cyanidine-3-O-rutinoside, where the average concentrations were 54.66, 141.08, 35.48, 494.08, and 478.66 μg/g dw, respectively. Similarly, the Spanish variety “Cuello Dama Blanca” combined the highest levels of chlorogenic acid, luteolin-7-O-glucoside, quercetin, and apigenin with 8.76, 17.9, 59.52, and 4.84 μg/g dw, respectively.

**TABLE 2:** Descriptive analysis and multivariate ANOVA of all studied variables over fig peels and pulps.

Variables		Mini	Max	Mean	*SD*	Dominance	ANOVA p-value
**Peel**							
Gallic acid		0	11.29	0.54	2.24	2	< 0.001
(+)-Catechin		0	24.06	5.89	5.95	24	< 0.001
(−)-Epicatechin		2.61	55.44	17.31	12.89	25	< 0.001
Chlorogenic acid		0	10.67	3.03	2.94	24	< 0.001
Quercetin-3-O-rutinoside		5.3	147.42	58.46	38.66	25	< 0.001
Quercetin-3-O-glucoside		2.52	35.58	11.48	7.76	25	< 0.001
Luteolin-7-O-glucoside		0	18.24	6.75	4.87	22	< 0.001
Quercetin		0	59.61	4.49	12.48	15	< 0.001
Apigenin		0	4.91	0.41	1.04	5	< 0.001
Cyanidin-3,5-diglucoside		0	495.76	48.58	109.91	16	< 0.001
Cyanidin-3-O-rutinoside		0	478.9	46.78	105.29	15	< 0.001
Pelargonidin-3-O-rutinoside		0	12.67	0.67	2.58	2	< 0.001
**Pulp**							
Gallic acid		nd	nd	nd	Nd	nd	–
(+)-Catechin		0	6.65	1.47	1.4	19	< 0.001
(−)-Epicatechin		1.25	19.06	5.23	4.03	25	< 0.001
Chlorogenic acid		0	4.84	0.77	1.09	19	< 0.001
Quercetin-3-O-rutinoside		0	26.85	1.89	5.16	17	< 0.001
Quercetin-3-O-glucoside		0	4.05	0.44	0.95	6	< 0.001
Luteolin-7-O-glucoside		0	4.5	0.21	0.89	2	< 0.001
Quercetin		nd	nd	nd	Nd	nd	–
Apigenin		nd	nd	nd	Nd	nd	–
Cyanidin-3,5-diglucoside		0	28.45	5.82	6.68	24	< 0.001
Cyanidin-3-O-rutinoside		0.94	34.43	9.01	8.67	25	< 0.001
Pelargonidin-3-O-rutinoside		nd	nd	nd	Nd	nd	–
**Effect**	**Wilks Lambda’s value**	**F**	**Hypothesis df**	**Error df**			**Significance**
Variety	0	477.23	560	1376.367			0
Fruit part	0	496075.72	20	79			0
Variety * Fruit part	0	464.37	440	1242.807			0

*nd, not detected; df, degree of liberty; F, refers to Fisher statistic; Sig., signification; Cyan, cyanidin; cy-3-r, Cyanidin-3-rutinoside; dominance, denotes the number of samples, where the phenolic compound was identified.*

In pulps extracts, (−)-epicatechin and cyanidin-3-O-rutinoside were the major compounds, which were identified in all samples at high levels:1.25–19.06 and 0.94–34.43 μg/g dw, respectively. Cyanidin-3,5-diglucoside were the third predominant compound that ranged from 0.81 to 28.45 μg/g dw, with a mean of 6.06 ± 6.71 μg/g dw, followed by (+)-catechin and chlorogenic acid (1.93 ± 1.29 and 1.01 ± 1.16 μg/g dw, respectively). However, luteolin-7-O-glucoside was detected in only two cultivars, “Chetoui” and “Palmeras,” with the following average concentrations: 0.75 ± 0.35 and 4.47 ± 0.04 μg/g dw, respectively. These results are, generally in agreement with those of Del Caro and Piga (del Caro and Piga, 2008), who used the same method on the Italian varieties “Mattalona” and “San Pietro.” These levels, mainly of (+)-Catechin, cyanidin-3-O-rutinoside, and luteolin-7-O-glucoside, are much higher compared with bananas, pears, and apples, however, similar to black grapes (Rothwell et al., 2013). Contrary to our findings, Palmeira et al. (2019) reported that rutin (quercetin-3-O-rutinoside) was the predominant PC in fig peel. In our samples, cyanidine-3,5-diglucoside and cyanidine-3-O-rutinoside were apparently predominant.

### Fourier Transform Infrare-Attenuated Total Reflectance Spectral Features

**FIGURE 1:**
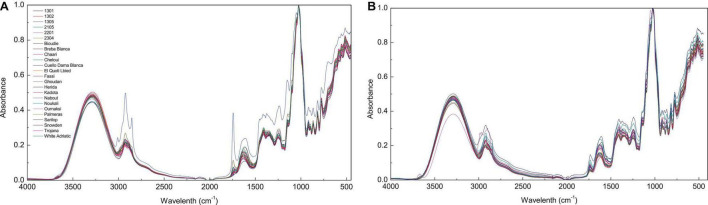
Spectra of different fig cultivars. FTIR spectra with absorbance value 450–4,000 cm^−1^ (before data pre-processing); **(A)**, Fig pulp spectra; **(B)**, fig peels.

Fourier transform infrared spectra of fig peel and pulp samples are shown in Figure 1. The bands observed between 4,000 and 450 cm^–1^ displayed six fingerprints around the following absorption regions: 3,700–3,000, 3,000–2,800, 1,775–1,725, 1,700–1,550, 1,500–1,300, and 1,175–940 cm^–1^ (Figure 1). The first region is most likely assigned to fibers, which are highly present in fresh figs. The broadband in this region is probably due to the O-H stretching vibrations arising from hydrogen bonding in cellulose. The absorbance at the region 3,000–2,800 cm^–1^ is most likely assigned to C-H, O-H, and NH3, which may be referred to carbohydrates, carboxylic acids, free amino acids, and phenolics (Schwanninger et al., 2004; Baranowska-Wójcik and Szwajgier, 2019; Palmeira et al., 2019). It is noteworthy that this band was divided into two peaks at 2,925 and 2,855 cm^–1^ (Bouafif et al., 2008). The first one is probably related to C-H stretching of a lipid’s methylene group. While the peak raised approximately at 2,855 cm^–1^ is possibly due to C-H stretching (symmetric) of CH_2_ from lipid acyl chains.

The peak at 1,775–1,725 cm^–1^ was associated to ester carbonyl band stretching (C=O). This vibration region is within the range of 1,800–1,700 cm^–1^, which is most probably correlated to the elongation of C=O of the ester type carboxylic (Oh et al., 2005; Bouafif et al., 2008). In case of the pulp samples, this vibration is most likely linked to the lipids contained in the fruit seeds (Vongsvivut et al., 2013). However, in the previous study by Terpugov et al. (2016) the peak was attributed to proteins. The vibration in the region of 1,700–1,550 cm^–1^ is typically originated to stretching band of carbonyl groups C=O and C=C (Tahir et al., 2017). The vibration bands in the region of 1,500–1,300 cm^–1^ corresponds to phosphodiester groups. This band is probably a result of several weak peaks that could not be differentiated among investigated samples. According to previous studies, this absorption band usually includes, among others, a vibration around 1,392 cm^–1^ that is most likely assigned to carbohydrates, fatty acids, or amino acids side chain (Vongsvivut et al., 2013), the 1,315 cm^–1^ vibration is associated with CH_2_ rocking (Schwanninger et al., 2004), and a vibration approximately at 1,155 cm^–1^, which is a result of C–O stretching (Vongsvivut et al., 2013). Finally, the vibrations in the regions 940–1,175 cm^–1^ marks a very strong and sharp peak, which is probably assigned to C–OH group as well as the stretches C–C and C–O in the carbohydrate structure and C–O in the phenol. In this region, quercetin-3-O-rutinoside was reported to record the highest vibration intensity around the wavenumber of 1,149 cm^–1^ (Paczkowska et al., 2015). It is noteworthy that this compound was found to be the major PC in all samples particularly in peel extracts (Table 2). The vibration at 1,149 cm^–1^ was assigned to C-C-H bending in benzene and dihydroxyphenyl aromatic rings (Paczkowska et al., 2015).

**FIGURE 2:**
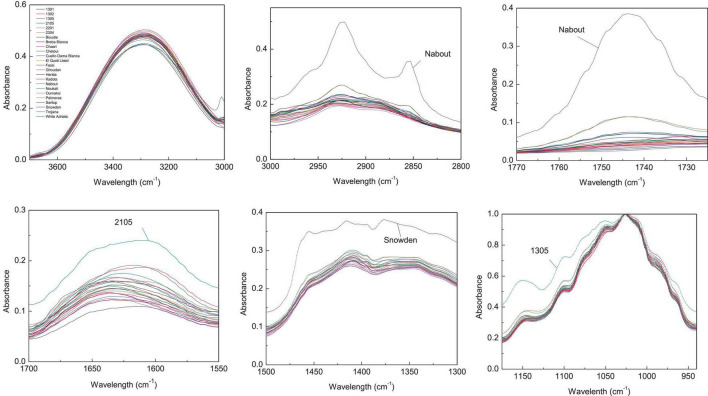
Integrated ATR spectra of the major wavenumbers in pulp samples. Each sample IR represent the mean of 3 spectra, which correspond each to the accumulation of 128 scans using a nominal resolution of 4 cm^−1^.

**FIGURE 3:**
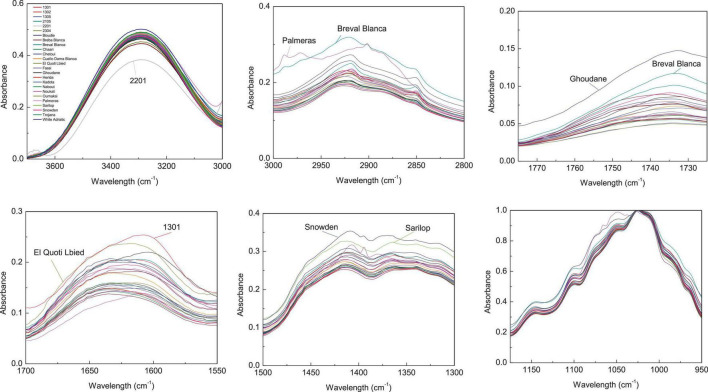
Integrated ATR spectra of the major wavenumbers in peels samples. Each sample IR represent the mean of 3 spectra, which correspond each to the accumulation of 128 scans using a nominal resolution of 4 cm^−1^.

Since the entire spectra of screened samples present a high overlapping level, the abovementioned major vibration range was plotted separately in Figures 2, 3 for both pulp and peel extracts, respectively. For pulp extracts, the bands around 1,775–1,725, and 3,000–2,855 cm^–1^ were remarkably clear for the variety “Nabout,” which recorded the highest absorbance intensity. In the bands corresponding to proteins (1,700–1,550 cm^–1^), organic acids (1,500–1,300 cm^–1^), and phenols (1,175–940 cm^–1^), the higher absorptions and integrated areas were recorded by “2201,” “Snowden,” and “1305,” respectively. These cultivars have combined, as previously mentioned, the promising levels of PCs, mainly (+)-catechin, quercetin-3-O-rutinoside, and quercetin-3-O-glucoside (Table 2). This makes sense, since these compounds record high vibration intensity at the wavenumber around 1,034 cm^–1^, which is mainly attributed to C–O stretching between mannopyranosyl and glucopyranosyl aromatic rings (Robb et al., 2002; Paczkowska et al., 2015).

Regarding peels extracts (Figure 2), the cultivars ‘Palmeras’ and ‘Breval Blanca’ revealed the highest absorption in the region of 3,000–2,800 cm^–1^. ‘Breval Blanca’ and ‘Ghoudane’ showed the highest absorption at the ester vibration region (1,775–1,725 cm^–1^), whereas, the cultivars ‘1301’ and ‘EL Quoti Lbied’ had the highest vibration in the proteins’ region (1,700–1550 cm^–1^). The vibration at the region of 1,500–1,300 cm^–1^ was superior in ‘Snowden’ compared with the other cultivars. In the phenols’ region (1,175–940 cm^–1^) ‘Chaari’ had the highest vibration intensity. Obviously, some dissimilarities between the both fingerprinting techniques are due to the fact that phenolics compounds revealed by HPLC-DAD could not totally explain the pattern yielded by FTIR fingerprinting between peel and pulp (Harvey et al., 2009).

### Results of Partial Least Squares Regression Models

Partial least square is a particular method because it can construct predictive models with highly collinear, noisy, and numerous factors, and also at once model several targeted variables (Wold et al., 2001). The robust model must combine a high *R*^2^, low RMSE, and a minimum number of latent variables (LVs), if at all possible less than ten (Liang et al., 2016; Table 3).

**TABLE 3:** Calibration and cross-validation results of multivariate models developed by using attenuated total reflectance Fourier transform infrared (ATR-FTIR) spectra in the regions of 1,175–940 cm^–1^ in both fig peel and pulp.

Phenolic compounds (μg g^–1^)	Peel extracts							Pulp extracts						
	LV	R^2^-Cal	R^2^-Val	RMSE-Cal	RMSE-Val	RPD-Cal	RPD-Val	LV	R^2^-Cal	R^2^-Val	RMSE-Cal	RMSE-Val	RPD-Cal	RPD-Val
((+)-Catechin)	5	0.97	0,76	0.73	2.49	2.38	1.86	8	0.85	0.81	0.9	1.9	2,52	2.23
(−)-Epicatechin	5	0.95	0.84	0.9	1.14	2.14	2.24	7	0.94	0.7	0.9	1.05	2.72	1.75
Chlorogenic acid	6	0.92	0.89	0.91	0.12	3.47	2.56	6	0.91	0.84	0.5	2.05	2.47	2.03
Quercetin-3-O-rutinoside	7	0.95	0.83	1.05	1.04	2,95	2.44	6	0.98	0,87	0.46	1.21	3.21	2.36
Quercetin-3-O-glucoside	7	0.87	0.74	0.97	1.21	3.72	2.3	–	–	–	–	–	–	–
Quercetin	6	0.99	0.83	0.82	1.09	2.44	2.62	–	–	–	–	–	–	–
Cyanidin-3,5-diglucoside	6	0.99	0.75	0.07	1.83	4.47	2.15	8	0.95	0.81	0.5	1.08	4.13	2.49
Cyanidin-3-O-rutinoside	8	0.96	0.86	0.04	1.54	3.84	2.43	7	0.96	0.88	0.6	0.23	4.21	2.67

*R^2^-Cal, coefficient of determination of calibration; RMSE-Cal, root mean square error of cross-validation.*

*R^2^-Val, coefficient of determination of validation; RMSE-Val, root mean square error of cross-validation.*

*LV, latent variable (orthogonal factors that provide maximum correlation with dependent variable).*

*RPD-Cal, residual predictive deviation of calibration; RPD-Val: residual predictive deviation of cross-validation.*

Partial least square regression models were built using the FTIR-ATR spectra set both in jack-knifing cross-validation and prediction set. These models were performed using IR data in the vibration region of 1,175–940 cm^–1^, corresponding to the C–OH group and the stretches C–O in the phenol structure. Within this region, the vibration band 1,540–1,175 cm^–1^ corresponds to flavanol and phenol (deformations of O–H, C–O, C–H, and C=C) (Masek et al., 2014; Nickless et al., 2014). It is noteworthy that gallic acid and pelargonidin-3-O-rutinoside were not involved in the PLSR for peel samples since these compounds were detected only in few cultivars. Similarly, quercetin-3-o-rutinoside and luteolin-7-o-glucoside were scarcely detected and then excluded in PLSR analysis.

**FIGURE 4:**
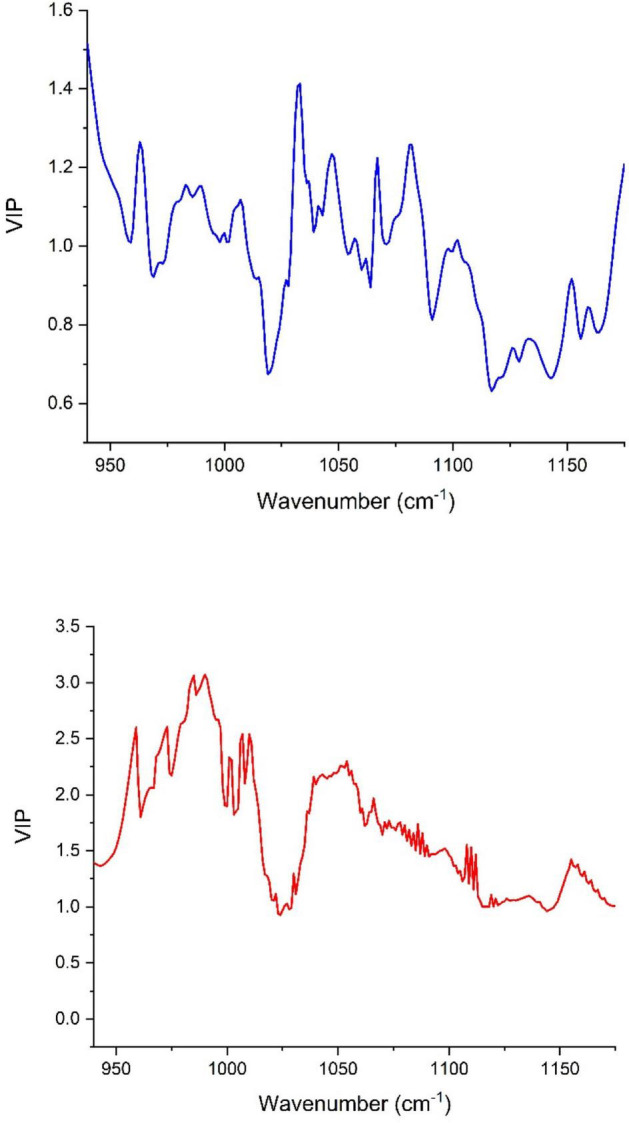
Variables importance in projection (VIP) plot showing the contribution of each wavenumber in the PLSR model total variance for both peel (blue) and pulp (red).

The number of LVs revealed by the models oscillated between 5 and 8 for the peel samples, whereas it was in the range of 6–8 in the pulp samples. Most of the number of LVs obtained by models built using pulp extracts were smaller than nine, henceforth demonstrating that the constructed models were not overfitting the data (the data used in observations fitting based on the learning set might not be practical to fit new observations) (Notions, 2010). In Figure 4, we see the contribution [variables importance in projection (VIP)] of each wavenumber within the wavenumber range of 1,175 and 940 cm^–1^ where the samples were scanned using the FTIR-ATR. The high value of VIP score indicates a great contribution to the model building. For peel samples, in order of importance, the following wavenumbers 940, 1,030, 1,081, 936, and 1,067 cm^–1^ had the highest VIP scores (> 1.2) and therefore, the main contribution to the built model total variance. Thus, the bands at 940 and 936 cm^–1^ are most likely assigned to C–C and C–O vibration (Movasaghi et al., 2018), while the peak at 1,030 cm^–1^ is attributed to C–O vibration (Paluszkiewicz and Kwiatek, 2001). At 1,081 cm^–1^, the peak is probably assigned to the ring stretching mixed strongly with CH in-plane bending (Chiang et al., 1999). The absorbance at 1,067 cm^–1^ is assigned to (C=O)–O– stretching, which is most likely an ester spectral peak (Armenta et al., 2005; Dong et al., 2013). On the other hand, the main loading for pulp samples were observed around the following wavenumbers 536, 541, 509, 524, 558, and 561 cm^–1^, which are most likely originated from strong deformation of C-C-C and C-C-O. These bands displayed the greatest VIP scores (>2.5) indicating a highest contribution to the model total variance. It should be noted that the VIP scores were much greater for the model built based on the pulp extracts compared with the peel samples prediction model (Figure 4).

Partial least square regression models statistics showed various levels of accuracy depending on targeted variables (PCs) and their partitioning in both fruit parts (peel and pulp). For peel samples, a good calibration model was achieved. Thus, the statistics displayed a coefficient of determination (*R*^2^-Cal) ranging between 0.87 and 0.99, along with root mean square error (RMSE-Cal) values in the range of 0.73 and 1.05. Similarly, the R^2^ for cross-validation was important for all PCs and was oscillating between 0.75 and 0.89 coupled with relatively low RMSE that was in the range of 0.12 and 2.49.

**FIGURE 5:**
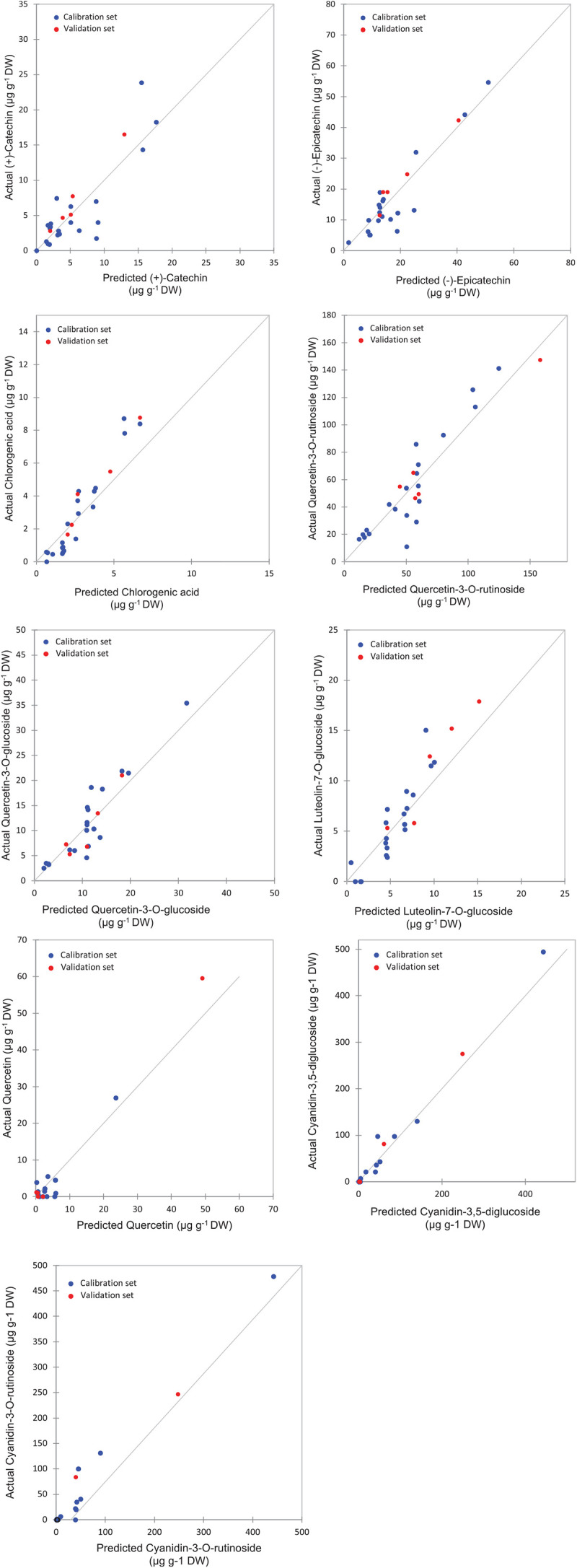
Correlation plots for the prediction of phenolic compounds in the peel samples using PLSR based on the FT-IR spectra.

**FIGURE 6:**
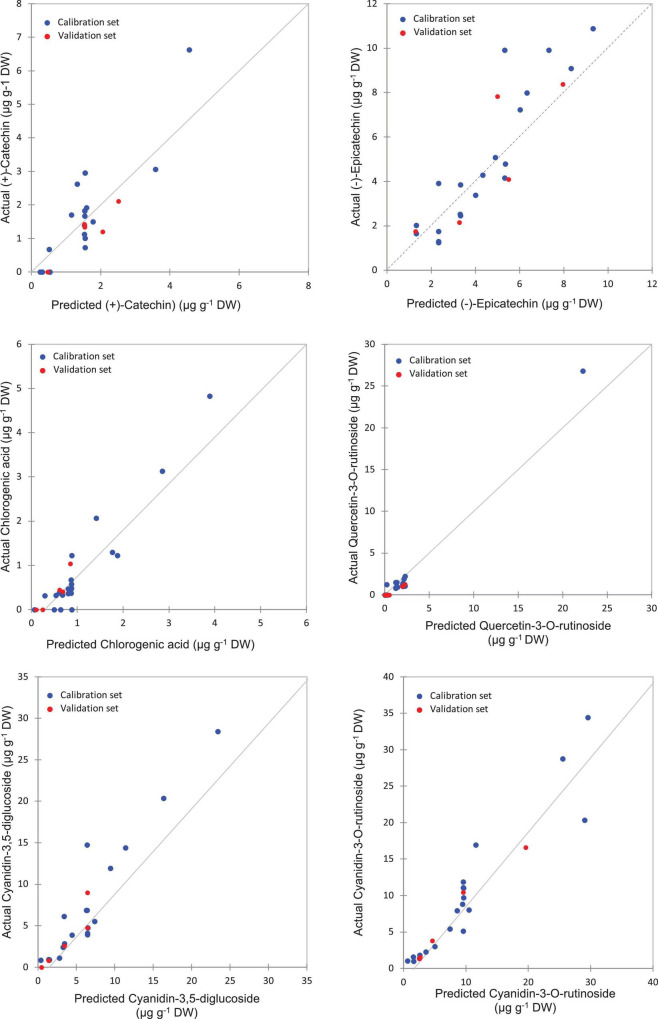
Correlation plots for the prediction of phenolic compounds in the pulp samples using PLSR based on the FT-IR spectra.

The highest levels of prediction were particularly observed for chlorogenic acid (*R*^2^-Val = 0.86, RMSE-Val = 0.12), followed by (−)-epicatechin (*R*^2^-Val = 0.84, RMSE-Val = 1.14) (Table 3). Scatter plots for the reference (*y*-axis) vs. predicted values (*x*-axis) for phenolic acids and flavonoids over the peel powders using FTIR-ATR spectroscopy shown in Figures 5, 6, displayed the accuracy of predicting these compounds. The models constructed for pulp samples displayed good calibration and validation statistics (Table 3 and Figure 6). Thus, the *R*^2^ and RMSE were generally similar to those found in models constructed based on the peels’ extracts. In calibration models, the *R*^2^ values ranged between 0.85 and 0.98 along with RMSE values in the range of 0.46 and 0.9. For the validation models, *R*^2^ and RMSE were in the range of 0.7–0.88 and 0.23–0.05, respectively. The highest performance of prediction was mainly observed for quercetin-3-O-rutinoside and cyanidin-3-O-rutinoside of which the *R*^2^-Val were higher than 0.87 with the respective RMSE of 0.23 and 1.21 (Table 3). RPD has different interpretations in the literature.

Hence, according to Morgan et al. (1994), an RPD value in the range of 2.5 and 3 seems adequate for screening and an interval of 3–5 is assumed better for quality assurance. On the other hand, Chang et al. (2001) assumed that the RPD greater than 2 is sufficient for a high throughput resolution, while the RPD < 1.4 may not lead to a reliably prediction. The results in Table 3 showed the RPD in the range of 2.14–3.84 and 1.86–2.62 for peel samples calibration and validation, respectively, whereas, for pulp samples it was in the following ranges 3.52–4.21 and 1.75–2.67, respectively, for calibration and validation. Overall, the abovementioned value seemed acceptable and suggest satisfactory throughput resolution of phenolic acids and flavonoids over both fruit parts. It is noteworthy that quercetin-3-O-glucoside and cyanidin-3,5-diglucoside alongwith cyanidin-3-O-rutinoside, displayed the highest RPD values for calibration step, which were generally greater than 3. For calibration, the same compounds displayed acceptable level for validation step with value greater than 2 for both fruit parts, except quercetin-3-O-glucoside which was not detected over pulp extracts.

Over all, both fruit parts revealed a reliable throughput resolution in predicting the concentrations of phenolic acids and flavonoids in different fig samples. Although the prediction of some compounds seemed to be slightly lower but remains good, since they were detected at a very minor levels and were not identified in all herein involved samples.

In raw material with high moisture content, such as figs (Farahnaky et al., 2009), the use of mid infrared spectroscopy is often reliable since it presents a low overlapping probability with the important bands associated with the measured property (Cozzolino et al., 2021). Based on the findings, the FTIR-ATR spectroscopy could be recommended as a simple and direct technique to assess the phenolic composition of figs, which does not require a tremendous sample pre-treatment or processing allowing for the development of a high throughput analytical method.

### Partial Least Squares Discriminant Analysis

**FIGURE 7:**
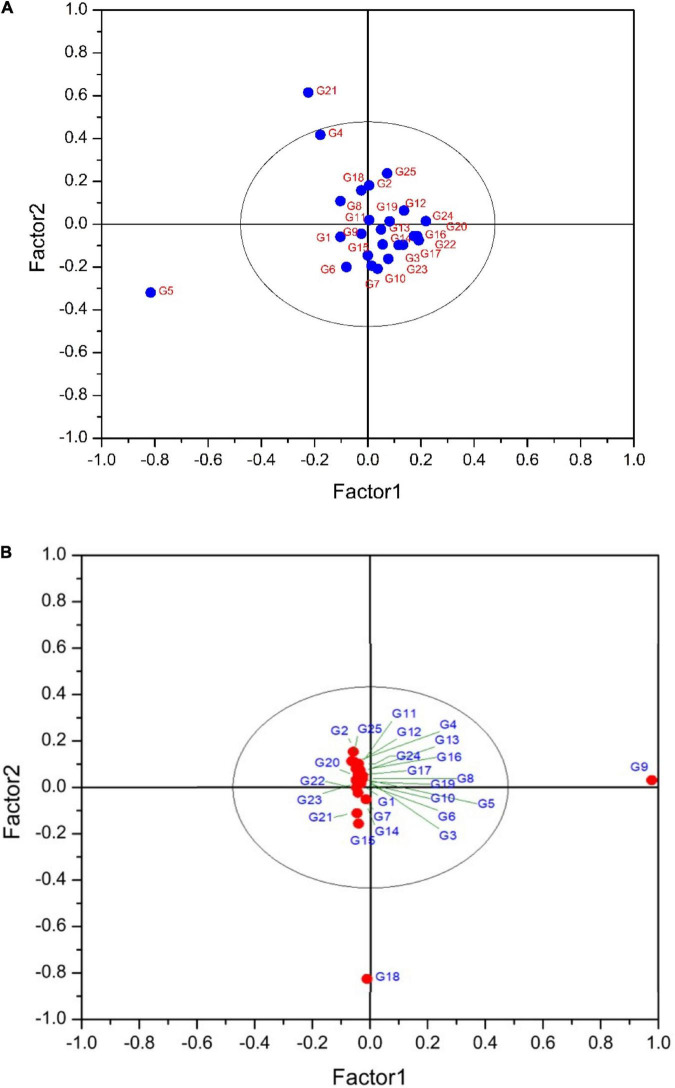
**(A)** PLS-DA plot showing the distribution of scanned peel samples. The variance explained by the factor1 and 2 are 77.40 and 15.44%, respectively. G1 to G25 refer to the cultivars codes as given in the Table 1. **(B)** PLS-DA plot showing the distribution of scanned pulp samples. The variance explained by the factor1 and 2 are 80 and 10.78%, respectively. G1 to G25 refer to the cultivars codes as given in the Table 1.

Partial least squares discriminant analysis is a linear classification approach that combines the properties of PLSR with the high discrimination feature of a classification method. The principal advantage of Partial Least Squares Discriminant Analysis (PLS-DA) is LVs, which model the main sources of variance within the data, and represent linear combinations of the original variables. Therefore, it allows the graphical presentation and understanding of the special distribution of the different data patterns and relations by LV scores and loadings (Ballabio and Consonni, 2013). Figures 7A,B shows that the data are not strongly clustered, particularly for the pulp samples that showed a strong overlapping around the plot origin. Based on Table 4, cyanidin-3,5-diglucoside and cyanidin-3-O-rutinoside had the highest loadings on the first and second factor for both fruit parts. This is due to the fact that these compounds are the predominant anthocyanins in fig peel and pulp, as reported by Russo et al. (2014). The distribution of peel samples, revealed one main cluster, with two cultivars, classified each as a single item (Figure 7A). Similarly, the pulp samples distribution displayed a single main cluster, with high overlapping intensity. Two cultivars were highly distinguished from the agglomerated samples (Figure 7B). The low overlapping level observed in the PLS-DA of peel samples suggest the use of this part of the fruit in future studies as they may offer better discrimination on fig tree cultivars. This makes sense, since the fig peels were reported to held the high levels of PCs compared with the pulp (del Caro and Piga, 2008; Oliveira et al., 2009; Qin et al., 2015).

**TABLE 4:** Loadings of phenolic compounds in the partial least squares discriminant analysis (PLS-DA) factors for both peel and pulp extracts.

	Peel		Pulp	
	Factor 1	Factor 2	Factor 1	Factor 2
Gallic acid	0.48	0.78	–	–
(+)-Catechin	15.4	10.26	0.11	–0.03
(−)-Epicatechin	20.28	–11.33	–0.89	3.98
Chlorogenic acid	1.11	–3.36	–0.59	0.85
Quercetin-3-O-rutinoside	–30.07	–34.96	–1.27	1.98
Quercetin-3-O-glucoside	6.08	–6.53	–0.51	–0.80
Luteolin-7-O-glucoside	3.20	0.39	–0.24	–0.48
Quercetin	11.21	6.02	–	–
Apigenin	0.59	0.99	–	–
Cyanidin-3,5-diglucoside	153.04	–207.32	–1.30	8.72
Cyanidin-3-O-rutinoside	137.46	–188.69	–2.31	11.34
Pelargonidin-3-O-rutinoside	2.369	–3.81	–	–

## Conclusion

This study is the first work attempting to examine the ability of FTIR-ATR spectroscopy combined with chemometrics to predict phenolic acids and flavonoids in fresh figs, with regard to their partitioning between the fruit peel and pulp. The results demonstrated the great potential of such simple and rapid technique to predict the PCs in the powder samples, with a satisfactory throughput resolution. All PCs in both fruit parts displayed high levels of prediction in both calibration and validation models. Although the prediction of some few compounds seemed to be slightly lower but still remains good, since they were detected at a minor level and were not identified in all herein involved samples. Significant differences were observed between the models built for the two fruit parts. Similar divergence was observed based on the distribution revealed by PLS-DA, where the highest scores were captured by cyanidin-3,5-diglucoside and cyanidin-3-O-rutinoside. Therefore, the FTIR-ATR spectroscopic technique represents, particularly when combined with chemometric approach, a convenient alternative in terms of time and chemical inputs saving for routine analyses of large raw material sample length. This approach can be considered as an affordable methodology, but one of the limitations of this work is that a larger sample length is required and further validation must be performed using samples from other varieties and origins.

## Data Availability Statement

The original contributions presented in the study are included in the article/supplementary material, further inquiries can be directed to the corresponding author.

## Author Contributions

LH: supervision, conceptualization, methodology, software, writing—original draft, resources, formal analysis, data curation, resources and visualization and review and editing. RR: data curation, resources, and visualization. YB: data modeling, software, and data inspection. All authors contributed to the article and approved the submitted version.

## Conflict of Interest

The authors declare that the research was conducted in the absence of any commercial or financial relationships that could be construed as a potential conflict of interest.

## Publisher’s Note

All claims expressed in this article are solely those of the authors and do not necessarily represent those of their affiliated organizations, or those of the publisher, the editors and the reviewers. Any product that may be evaluated in this article, or claim that may be made by its manufacturer, is not guaranteed or endorsed by the publisher.
